# A novel method to identify gene interaction patterns

**DOI:** 10.1186/s12864-021-07628-9

**Published:** 2021-06-10

**Authors:** Xinguo Lu, Fang Liu, Qiumai Miao, Ping Liu, Yan Gao, Keren He

**Affiliations:** 1 College of Computer Science and Electronic Engineering, Hunan University, Lushan Nan Road, Changsha, 410082 China; 2 Hunan Want Want Hospital, Renmin Zhong Road, Changsha, 410006 China

**Keywords:** Module network, Protein-protein interaction, Gene communities

## Abstract

**Background:**

Gene interaction patterns, including modules and motifs, can be used to identify cancer specific biomarkers and to reveal the mechanism of tumorigenesis. Most of the existing module network inferencing methods focus on gene independent functional patterns, while the studies of overlapping characteristics between modules are lacking. The objective of this study was to reveal the functional overlapping patterns in gene modules, helping elucidate the regulatory relationship between overlapping genes and communities, as well as to explore cancer formation and progression.

**Results:**

We analyzed six cancer datasets from The Cancer Genome Atlas and obtained three kinds of gene functional modules for each cancer, including Independent-Community, Dependent-Community and Merged-Community. In the six cancers, 59(3.5%) Independent-Communities were identified, while 1631(96.5%) Dependent-Communities were acquired. Compared with Lemon-Tree and K-Means, the gene communities identified by our method were enriched in more known GO categories with lower *p*-values. Meanwhile, those identified distinguishing communities can significantly distinguish the survival prognostic of patients by Kaplan-Meier analysis. Furthermore, identified driver genes in the gene communities can be considered as biomarkers which can accurately distinguish the tumour or normal samples for each cancer type.

**Conclusions:**

In all identified communities, Dependent-Communities are the majority. Our method is more effective than the other two methods which do not consider the overlapping characteristics of modules. This indicates that overlapping genes are located in different specific functional groups, and a communication bridge is established between the communities to construct a comprehensive carcinogenesis.

**Supplementary Information:**

The online version contains supplementary material available at (10.1186/s12864-021-07628-9).

## Background

Cancer is a complex disease that threatens human health, and it is mainly driven by the accumulation of a series of genetic changes that can be detected by various high -throughput sequencing technologies [1, 2]. With the development of high- throughput next generation sequencing (NGS) [3] technology, many large genomics projects have produced and accumulated a large amount of genomics data, such as The Cancer Genome Atlas (TCGA) [4] and the Catalogue Of Somatic Mutations In Cancer (COSMIC) [5]. The rapid accumulation of massive multi-type data provide an unprecedented opportunity to capture cancer formation and progression [6, 7]. According to the tumorigenesis, the alterations of pathological gene expression and the progression of cancer involve many factors [8, 9], such as metastasis development, invasion, proliferation, angiogenesis and other contributing factors [10]. If we can accurately understand the pathogenesis and process of tumors, the corresponding treatment methods can be adopted to avoid the serious damage caused by tumors.

Due to the high dimensionality and heterogeneity of cancer data, researchers address an enormous challenge to obtain mutant biomarkers that promote the process of tumorigenesis proliferation from these massive high-throughput data [11–13]. Recent studies have shown that the detecting modular network brings new prospects for the development of biological mechanisms and tumors [14, 15]. Many module network approaches have been proposed for gene module identification in the past decades. These methods can be divided into three categories, including heuristic clustering module identification method, model-based clustering method and the module network inference method based on gene-based network reconstruction, which depend on different biological assumptions and intuitions. The original module network learning algorithm relies on greedy heuristic algorithm, which was inspired by this viewpoint that co-expressed genes are likely to have similar regulatory patterns and may have the same driver or regulatory genes. For example, Eisen et al. applied a pairwise average-linkage cluster analysis approach which is a form of hierarchical clustering to gene expression data verified that Clusters of coexpressed genes tend to be enriched for specific functional categories [16]. Although these methods have had an enormous influence, their statistical properties are generally not well understood and important parameters such as the number of clusters are not determined automatically [17]. Therefore, the attention has subsequently shifted to model-based clustering methods, Dahl et.al proposed a approach to obtain a point estimate of the clustering based on the least squares distances from posterior probabilities of two gene clusters [18]. The method groups genes with equal potential variables that control expression, and combines the least squares distances to automatically estimates the number of clusters. Anagha joshi et al. proposed a model clustering method based on Gibbs sampler [17]. This method uses Bayesian method and Gibbs sampling process to iteratively update the cluster assignment of each gene and condition. These methods assumes that the data is generated by a mixture of probability distributions (one for each cluster) and explicitly considers the noise of gene expression data. It allows for a statistical estimate of the generated clusters and gives a formal assessment of the expected number of clusters [17].

In recent years, it has been discovered that module network inference can be combined with gene-based network reconstruction approachs [19, 20]. This methodological work complements research that focuses only on applying module network mehods to provide new biomedical and biological insights. For instance, Eric Bonnet et al. proposed a multi-omics module network reasoning method called Lemon-Tree [21], which uses one or more Gibbs samples to infer the initial module from gene expression data, and then use a spectrum edge clustering algorithm to establish a consensus module of genes. Although these methods have achieved good results, these proposed methods of module network recognition based on gene network reconstruction all insist that gene modules are independent and unrelated with each other. Contrary to this point of view, in real biological networks, gene modules may have overlapping characteristics [22]. For example, overlapping characteristics for gene GATA3 and TP53 in different functional pathways in breast cancer in which GATA3, TP53 and PIK3CA regulate the process of neuron apoptotic while gene GATA3, TP53 and BRCA2 response to gamma radiation [23]. This reveals that these genes with overlapping features have different functional roles in different communities. By obtaining the effects of overlapping factors in different gene communities, additional biomarkers may be identified which provide an effective complement to understand cancer progression. Therefore, we import the concept of overlapping community detection to module networks inference. The method is remarkably superior in deducing functional units and overlapping genes which can play different roles in the cell by taking part in several processes.

**Fig. 1:**
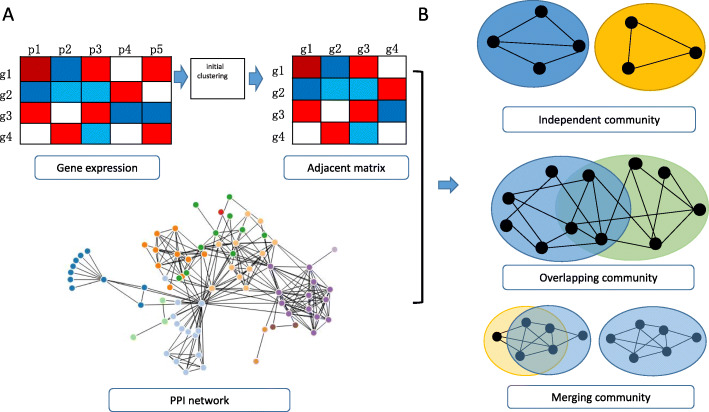
Schematic of our method. **a** Illustration of the relationship of two gene with protein-protein interaction (PPI) network and gene expression. Genes in gene expression matrix are pre-processed by gene differential expression analysis to select candidate gene subset. The generation of adjacency matrices is by running one or more instance of initial community procedure. Then, the PPI network is combined to the association between genes. **b**The gene community is captured by these three models including Independent-Community, Dependent-Community and Merged-Community

Here we propose a method to identify cancer function modules based on the concept of overlapping community detection (Fig. 1). The aim of this study was to reveal the overlapping characteristics of gene modules and explore the regulatory relationship between overlapping genes and modules by integrating comprehensive genomic data including gene expression data, copy number data (CNV) and protein-protein interaction (PPI) network data. We first used the Gibbs sampling model to generate initial clusters by clustering gene expression data, and then combined the PPI network with the initial clustering results to construct an interaction network. Finally, functional overlapping modules were detected from the network by overlapping community detection method. Considering overlapping scores, the gene community could be divided into three types: Independent-Community (IC), Dependent-Community (DC) and Merged-Community (MC). Experimental results indicate that ICs identified by our method have independent functions. Overlapping genes in DCs have diverse functions and bridge dependency among communities. The effect of MCs merge is found not only in the network structure but also in the corresponding known pathway function. In Go enrichment analysis, our method can identify more GO categories with lower *p*-values than other tools. In addition, the driver genes generated can accurately distinguish the tumor and normal samples. The identified communities can distinguish survival and prognosis of patients by Kaplan-Meier analysis.

## Results

### ICs identified for characterizing breast subtypes

We applied our method to each gene expression profile of six cancer datasets (see Table 1) from TCGA for community detection. The community was obtained based on the threshold of overlap score (*ω*) with 0.8 and the change of belonging factor of each node *a*_*iS*_ in each model. The number of Independent-Community and Dependent-Community is shown in Table 2. In Bladder Urothelial Carcinoma, we captured 193 communities that contain 12 ICs and 181 DCs. And 11 ICs and 208 DCs were found in Breast invasive carcinoma cancer, 12 ICs and 449 DCs in Colon adenocarcinoma, 7 ICs and 145 DCs in Esophageal carcinoma, 7 ICs and 294 DCs in Head and Neck squamous cell carcinoma. In addition, the total number of community in Kidney Chromophobe is 364 communities, including 10 ICs and 354 DCs. The Merge-Community was not uncovered here since the pair communities were merged into one community. The result shows that DCs were the majority of all communities, further illustrating the strong communication between communities.

**Table 1:** Cancer types and number of samples for tumor and normal tissues from TCGA database

Cancer Type	TCGA ID Data	No. Tumor samples	No. Normal samples
Bladder Urothelial Carcinoma	BLCA	407	19
Breast invasive carcinoma cancer	BRCA	485	8
Colon adenocarcinoma	COAD	288	41
Esophageal carcinoma	ESCA	185	11
Head and Neck squamous cell carcinoma	HNSC	522	44
Kidney Chromophobe	KICH	66	25

**Table 2:** The number of communities in different cancer

Cancer Type	Independent-Community	Dependent-Community	All Communities
BLCA	12	181	193
BRCA	11	208	219
COAD	12	449	461
ESCA	7	145	152
HNSC	7	294	301
KICH	10	354	364

**Fig. 2:**
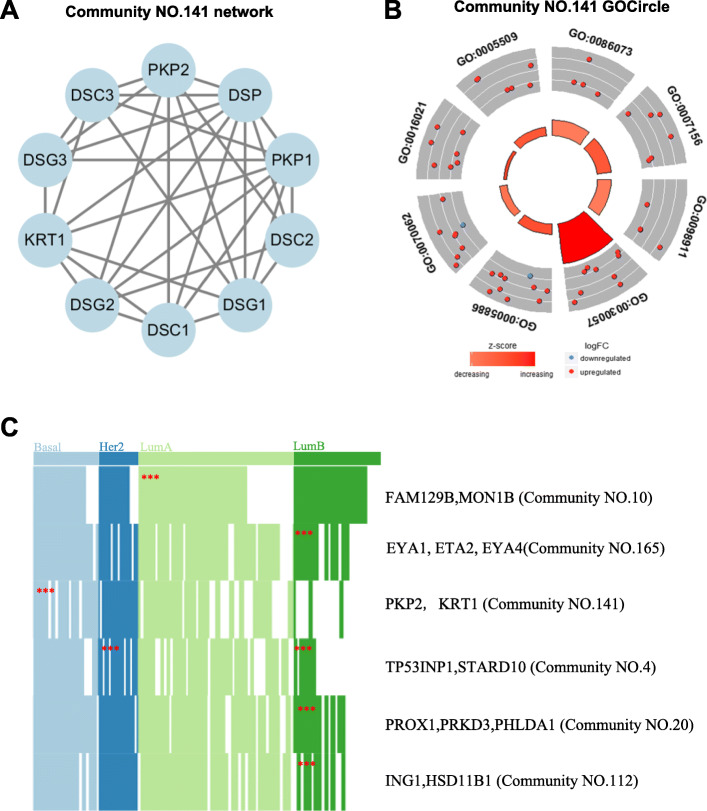
Independent-Community for TCGA Breast Cancer Dataset. **a** the network of IC141. **b** The GO enrichment result of Community NO.141. **c** the prediction of ICs in cancer subtypes

To clarify the results of the three models, we only analyzed breast cancer. Firstly, we validated the significance of functional interactions within IC communities with the Gene Ontology (GO) biology process (BP), cellular component (CC)and molecular functions (MF) with R package GOplot [24]. We captured 11 Independent-Communities for breast cancer. Figure 2a shows one Independent-Community network. The rest of Independent-Communities networks are shown in Additional file 1: Figure S1. The Independent-Community (Community NO.141) GO enrichment analysis is presented in the Fig. 2b. In terms of cellular component, the majority of genes are mainly enriched in plasma membrane process (GO:0005886, *p*-value = 1.536E-06), desmosome process (GO:0030057, *p*-value = 2.195E-23), extracellular exosome process (GO:0030057, *p*-value = 5.567E-5). In terms of biology process, bundle of His cell-Purkinje myocyte adhesion involved in cell communication process (GO:0086073, *p*-value = 2.128E-09), homophilic cell adhesion via plasma membrane adhesion molecules process and regulation of ventricular cardiac muscle cell action potential process (GO:0007156, *p*-value = 8.461E-9) are significantly enriched the genes in this community. In molecular functions category, genes are enriched in calcium ion binding process (GO:0005509, *p*-value = 1.490E-5) and integral component of membrane process (GO:0016021, *p*-value = 0.0189). It elucidated that the identified ICs are independently functional. Then, we validated the association of the communities with breast cancer subtypes (Luminal A (LumA) ∖ B (LumB), Her2 and Basal) (Fig. 2C and Additional file 1: Table S1)as well as their association with mutagenic processes independent of the subtyping through the mutation behaviour of the core genes with top degrees. We found that the EYA1/EYA2/EYA3 (Community NO.165), PROX1, PRKD3, PHLDA1 (Community NO.20) and ING1, HSD11B1 (Community NO.112) are significantly associated with the LumB subtype (p <0.01, Fisher’s exact test). The TP53INP1/STARD10 (Community NO.4) is significantly associated with the Basal subtype and Her2 subtype (p <0.01). The FAM129B/MON1B (Community NO.10) is prominently related to the LumA subtype (p <0.01). The PKP2/KRT1 (Community NO.141) is obviously enriched in the Basal subtype (p <0.01).

### Overlappying genes in DCs bridge dependency between communities

**Fig. 3:**
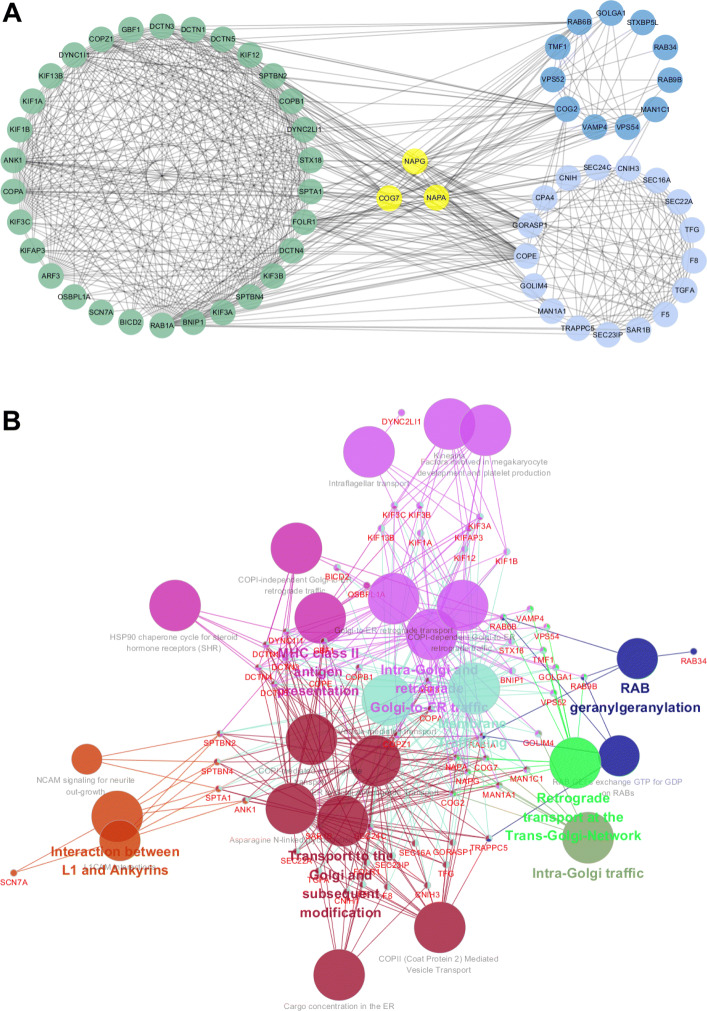
Dependent-Community for TCGA Breast Cancer Dataset. **a** The network of comunity 121,126 and 172. The green circle is DC126,the deongaree circle is DC 121 and the light blue circle is DC 172. The yellow circle is the overlap genes. **b** The analysis of the pathway in three communities.The large nodes represent pathways, small nodes represent genes, and edges represent gene express in pathway

In this model, we captured 208 Dependent-Communities which are completely shown in Additional file 2. The structure and property of overlapping characteristics of communities for Community NO.121, NO.126 and NO.172 are shown in Fig. 3. Cytoscape (version 3.5.1) [25] was used to analyze the overlapping structure among the communities. From Fig. 3a, we can see that the overlapping gene subset including COG7, NAPA and NAPG (yellow circle) bridged the Community NO.121 (deongaree circle), Community NO.126 (green circle) and Community NO.172 (light blue circle). Biologically, genes in the same community, such as Community NO.121, Community NO.126 and Community NO.172, often coincides with known functional modules and/or protein complexes [26]. The Reactome pathway analysis was performed to detect the specific function for these three communities respectively and the results are shown in Fig. 3b. Genes RAB1A, RAB34, RAB6B, RAB9B in Community NO.126 express in RAB geranylgeranylation pathway. CNIH3, F5, F8 etc genes in Community NO.172 locate in Cargo concentration in the ER pathway and so on. Since the overlapping genes are located in different communities with specific function, it indicates overlapping genes possess diverse functions. A gene can be relevant with more than one functional community (or cluster) [27, 28] in cancer patients and bridge dependency between communities. In Fig. 3b, the overlapping gene NAPA is in the Asparagine N-linked glycosylation pathway which corresponds to the Community 172 function. Meanwhile, NAPA is a link in Intra-Golgi traffic pathway which associates with the Community 121 function. In addition, NAPA plays a function in COPI-dependent Golgi-to-ER retrograde traffic pathway which binds with the community 126. And the overlapping genes COG7 and NAPG enriched in the pathway associated with these three DCs too (Fig. 3b and Additional file 1: Figure S2). The ratio of genes in each pathway and the genes contained in each pathway are shown in Additional file 1: Figure S2 and Additional file 3.

### MCs worked as a functional unit for communication

**Fig. 4:**
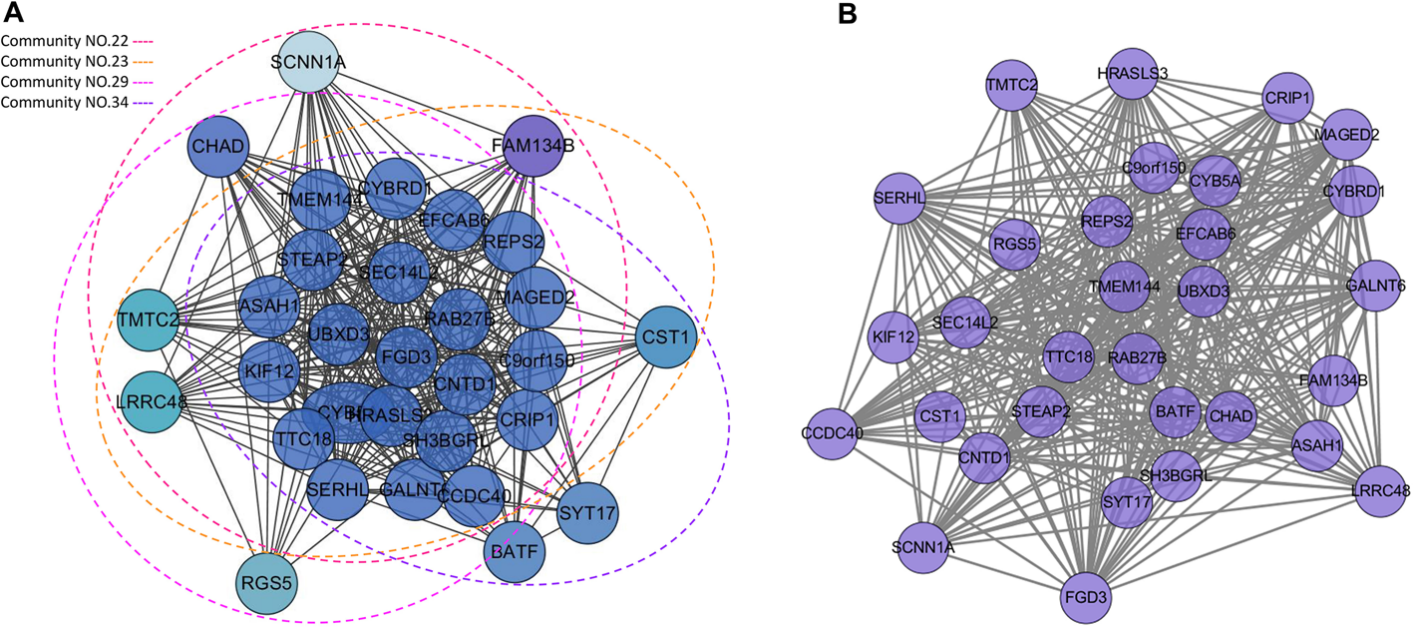
Merged-Community for TCGA Breast Cancer Dataset. **a** Four modules uncovered before fusion by Merged-Community for TCGA breast cancer dataset. **b** Four modules merged to one module

In order to further understand the integration mechanism of community, overlap score (*ω*) was set as 0.8 and 0.9 respectively. We captured 219 gene communities at the threshold of 0.8. While the threshold of 0.9, we obtained 237 gene communities. The MC model will merge the community with overlap score over 0.8, resulting in a reduction of 18 communities (Table 3). Subsequently, we calculated the overlap score for each pair of communities in 237 communities. We found that 33 communities out of these 237 communities were merged into 15 communities. The result of community merging is shown in Table 3. The detailed materials for community integration are shown in Additional file 4. Interestingly, these resulted communities are DCs due to the high frequency of interaction with other communities. For displaying an intelligible MCs result, we decipher a concrete case in Fig. 4. The overlap scores between Community NO.22 and Community NO.23, Community NO.22 and Community NO.29, Community NO.23 and Community NO.29, Community NO.23 and Community NO.34 are 0.89, 0.89, 0.85 and 0.82 (Fig. 4a), respectively, which are all higher than threshold (0.8). We merged them into one community which can perform as a common functional unit to action together (Fig. 4b). Jointly, the effect of merging can be seen not only in the network structure, but also in the corresponding known pathway function. The gene CST1 in Community NO.23 and Community NO.34 is significantly enriched in Salivary secretion pathway (*ω*=0.9) which is omitted from Community NO.22 and Community NO.29. Furthermore, the merged functional unit possesses the complementary function together in the result of merging gene CST1.

**Table 3:** The information of community merging

Types of community merging	The number of before merging community	The number of after merging community	The number of reduction community
Type 1	20	10	10
Type 2	6	2	1
Type 3	3	2	4
Type4	4	1	3
Total	33	15	18

### Prognostic predictor for cancer patients with survival analysis

**Fig. 5:**
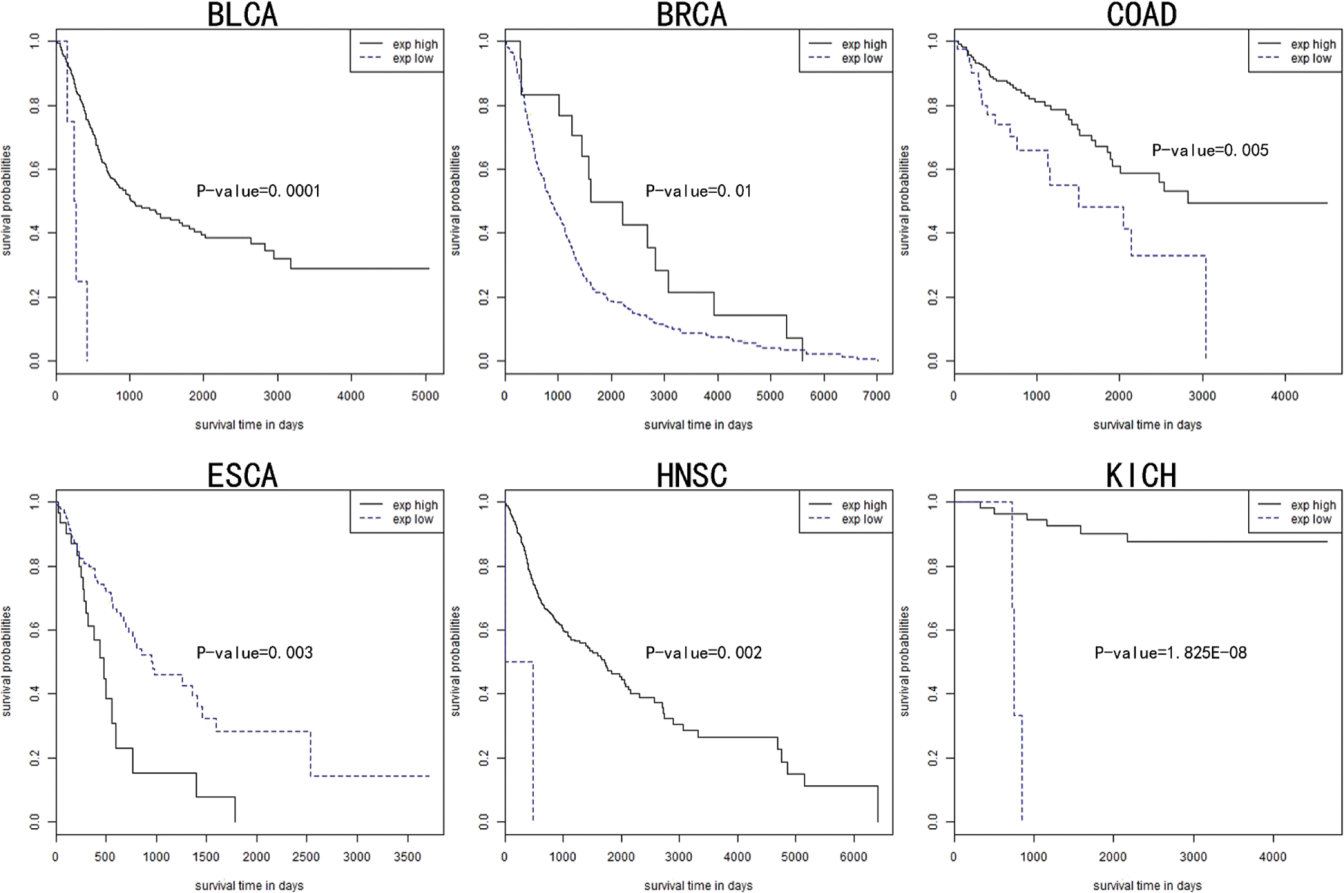
Kaplan-Meier survival analysis for patients based on the detected modules in each cancer

Gene and modules are associated with patient survival time in cancer [29]. In order to assess the biological relevance of community network, we analyzed the prognosis value of total communities by Kaplan-Meier survival analysis (see Methods). For each community, we predicted the patient survival time, using the clinical data and gene expression data. We found that both the Independent and Dependent Community divide the patients into two groups whose survival time is significantly different (*p*-value <= 0.01). Figure 5 shows the most significant survival associated community in six cancers. In BLCA, patients were significantly divided into two classes by the identified functional community (p = 0.0001). The community obtained from BRCA also reveals a significantly different survival time (*p*-value = 0.01). In COAD, we found the patients in the given community were divided into two groups and the survival time is significantly different (*p*-value = 0.005). In ESCA, the detected community is significantly associated with the survival time of patients (*p*-value = 0.003). In HNSC, the community can significantly classify patients according to the average expression values (*p*-value = 0.002). The most significant survival-related community was obtained from KICH (*p*-value = 1.825E-08). The analysis demonstrates that the six cancer communities could distinguish likely patient prognostic survival time according to their mean expression values of genes, with the statistical significance (p <= 0.01).

### Comparison for Lemon-tree,K-Means and our method

**Fig. 6:**
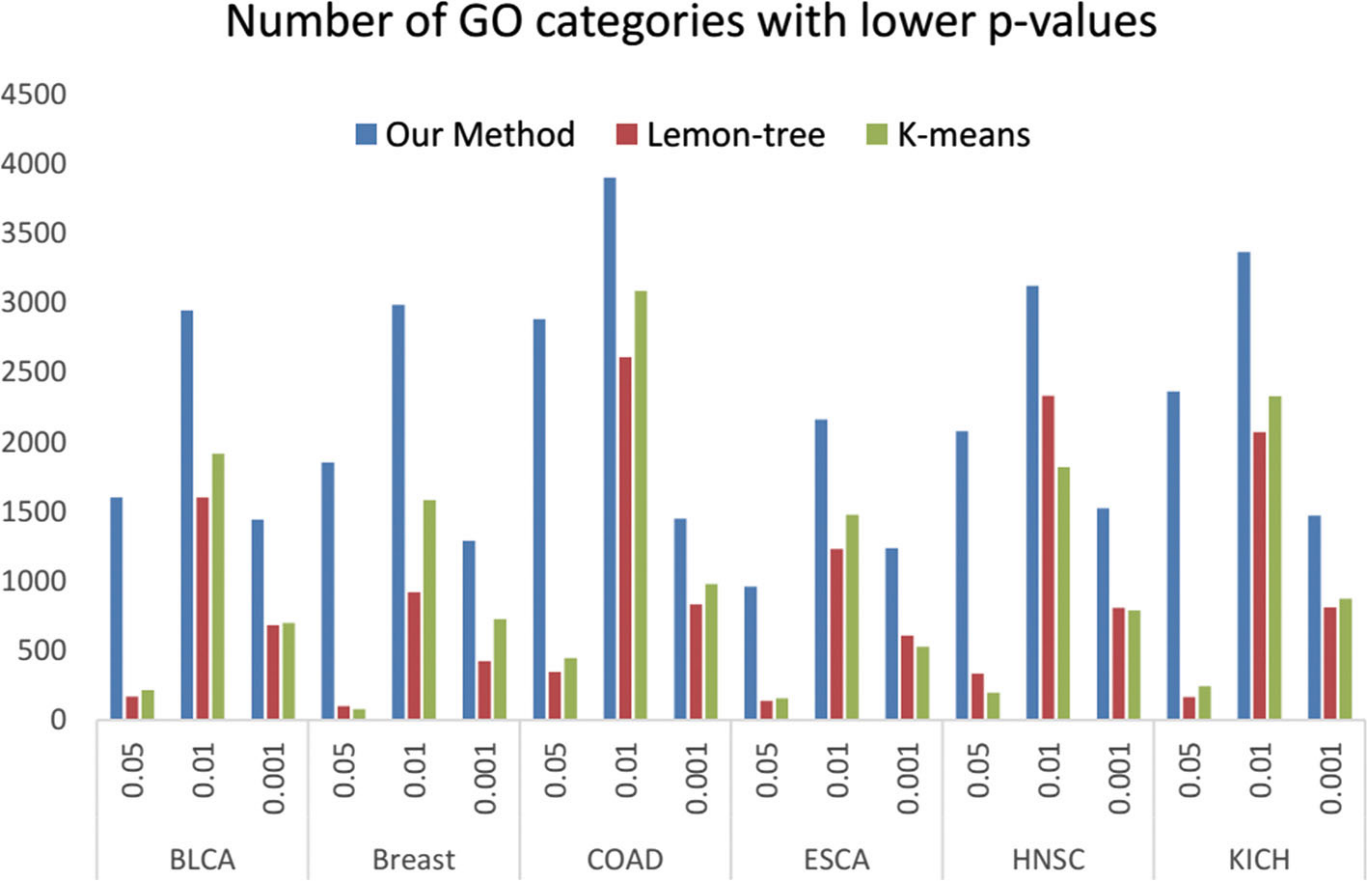
Gene Ontology (GO) enrichment of the co-expressed gene clusters,shows by counting the number of GO categories having a lower *p*-value

We compared our method with Lemon-Tree and K-Means over the same large-scale real cancer data set to evaluate the performance based on the Gene Ontology (GO) enrichment. To compare the Gene Ontology (GO) categories among the three approaches, we first obtained all common categories for each cancer by a given *p*-value threshold. Then, the highest score for each GO category was selected, and the number of GO categories with higher scores for K-Means, Lemon-Tree and our method was calculated. Finally, the sum of the scores for each GO category in each method is calculated (Fig. 6). This result indicates that the gene communities identified by our approach were enriched in more known GO categories with lower *p*-values than other methods, and our approach has a lower *p*-value in the global scope. Overall, our approach performs better than Lemon-tree and K-Means in terms of analyzing the GO enrichment.

### In silico validation

To verify the performance of our method in the classification of cancer versus normal samples for each cancer type, we captured the candidate driver genes by mutation frequency from TCGA data. Then the cancer driver genes were identified from the candidate driver genes. In our method, we selected the genes as candidate driver genes with mutation frequency fre >0.6. In the six cancers of BLCA, BRCA, COAD, ESCA, HNSC and KICH, the candidate regulator lists were comprised of 2077, 1100, 1910, 7312, 2230 and 9369 genes respectively. As the regulatory mechanism of communities or modules, the top 1% highest scoring genes of each community were selected as the final driver genes list. Finally, we obtained 745 cancer driver genes.

**Fig. 7:**
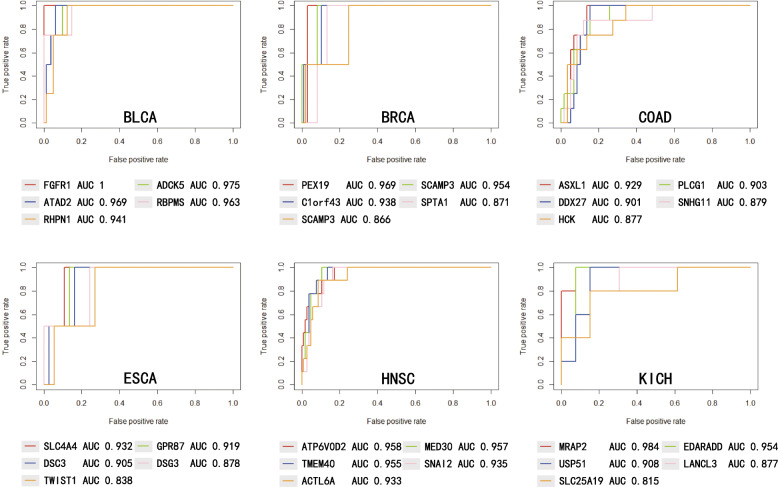
ROC Curves and AUC values for the top ten driver cancer-specific genes

For each cancer dataset, 80% of cancer versus normal samples were selested for training classifier, and the remaining datasets were used to test the classification performance. The results in The Cancer Genome Atlas data using 10-fold cross-validation are shown in Fig. 7. ROC curves and AUC values are shown for the top five cancer driver genes with the highest AUC performance for each cancer type. The genes PEX19 and SCAMP3 achieve the best AUC performance in BRCA (AUC = 0.969 and AUC = 0.954), ASXL1 and PLCG1 are the best predictors in COAD (AUC = 0.929 and AUC = 0.903), SLC4A4 and GPR87 in ESCA (AUC = 0.932 and AUC = 0.919), ATP6V0D2 and MED30 in HNSC (AUC = 0.958 and AUC = 0.957), MRAP2 and EDARADD in KICH (AUC = 0.984 and AUC = 0.954). The best performance was obtained in BLCA dataset. The top five drivers of cancer always obtained AUC >0.9, and the AUC for gene FGFR1 reaches 1 (Fig. 7).

The top-10 driver genes’ regulation scores in each cancer are shown is demonstrated in Additional file 1: Table S2. It proves that the low regulatory score of the driver genes can also accurately distinct tumor and normal samples.

MRAP2 is known to play a role in cancer [30]. the expression of MRAP2 is restricted to the adrenal gland and brain tissue, MRAP2 overexpression caused suppression of MC2R activation, and positive effects on signaling have been detected only at supraphysiological levels of ACTH. RBPMS has been reported as a part of the metastatic 79 gene characteristics observed in solid tumors [31]. Many studies implicated the RBPMS family of proteins in oocyte, retinal ganglion cell, heart, and gastrointestinal smooth muscle development. While SCAMP3, which is overexpression in the hepatocellular carcinoma [32], has been found that its modification with ubiquitin and its interaction with ESCRT coordinate the regulation of endosome pathway and affect the efficiency of receptor down-regulation [33]. SCAMP3 is a component of post-Golgi membranes, functions as a protein carrier and is critical for subcellular protein transportation. ASXL1 is widely expressed at low level in heart, brain, skeletal muscle, placenta, pancreas, spleen, prostate, small intestine, colon, peripheral blood, leukocytes, bone marrow and fetal liver. ASXL1 mutation is associated with some human cancer types such as leukemias and Bohring–Opitz syndrome [34]. GPR87 was suggested to contribute to the viability of human tumor cells and overexpression of GPR87 mRNA was detected in a number of malignant tumors, including lung cancer [35]. In addition, the high expression of TMEM40 is related to malignant behavior and tumorigenesis, which plays a key role in cell proliferation and apoptosis [36]. TMEM40 gene encodes a protein of 233 amino acids and is located on chromosome 3p25.2. TMEM40 silencing could dramatically suppressed cell proliferation, inhibited cell migration and decreased tumor growth. In invasive tumors, the overexpressed and amplified of ATAD2 has been reported [37], ATAD2 is mapped to chromosome 8q24.13, a genomic region frequently amplified in multiple cancer types,which is highly expressed in several different types of human tumors. while FSCN1, which is up-regulated by SNAI2 and promotes epithelial to mesenchymal transition in HNSC SNAI2 is key components for protein synthesis, which promotes epithelial to mesenchymal transition in HNSC [38], SNAI2 is usually upregulated and its high expression is associated with decreased cell-cell adhesion, increased motility and aggressive phenotype.

## Discussion

A large number of applications on high throughput data are applied for cancer diagnosis, clinical treatment and prognosis prediction [39]. In many cases, the biological networks are applied to study intertwined signaling cascades, such as gene co-expression network, metabolic networks and protein-protein interaction networks, which are helpful to explain the tumorigenesis [40]. Particularly, it is a matter of common experience that such networks seem to have community and motifs in them: subsets of vertices within which vertex-vertex connections are dense, but between which connections are less dense. Recent insights emerged from the clustering modules, a comprehensive understanding in many biological mechanisms has been revealed through probabilistic graphical models which considered regulated genes and their regulatory processes [14]. Although many functional independent modules are identified and the module inference is effective in some methods, they are unable to exploit the dependent relation between modules. However, in the real biological network, gene modules may pose the characteristic of overlap, i.e. some genes may belong to multiple groups, where a node might have more than one function.

Hence, we introduce the concept of overlapping community detection into modular network reasoning. Based on the concept of overlapping community detection in social networks, we develop a novel co-expressed network analysis framework that can efficiently construct and analyze the large biology networks. Three network inference models were constructed, including Independent-Community, Dependent-Community and Merged-Community, for representing the relationship between diverse gene communities. By utilizing these three models, our approach is proposed to obtain the gene community with functional overlapping characteristics through integrating gene expression data and PPI data. We applied the method in six cancer data to obtain the diverse composition of gene communities, the results of identified communities are shown in Table 2. According to Table 2 we can found that DCs are the majority of all communities, this illustrating the strong communication between communities. In order to clarify the results of the three models, breast cancer was analyzed in detail. First, for the Independent-Community, it can be seen from the results (Fig. 2b) that the identified integrated circuits have independent functions. Moreover, we also validated the association of the communities with breast cancer subtypes (Fig. 2c) as well as their association with mutagenic processes independent of the subtyping through the mutation behavior of the core genes with top degrees. we can find that for a given community, each mutation event across all the samples would markedly enriched in specific subtypes to effectually categorize patients. In addition, mutations in core genes do correlate with the progression of cancer subtypes. Remarkable relation among EYA1, EYA2 and EYA3 genes with the LumB subtype are in accordance with the previous finding that EYA genes play an important role in breast cancer growth and metastasis as well as directing cells to the repair pathway upon DNA damage [41, 42]. The community containing TP53INP1 is strongly mutated in the Her2 and LumB subtypes (p <0.01) mainly because of mutations in TP53INP1. Next, for the Dependent-Community, we find Biologically, genes in the same community often coincide with known functional modules and/or protein complexes [43]. And our result (Fig. 3b) shows that overlapping genes possess diverse functions and bridge dependency among communities. Moreover, overlapping characteristics for gene TP53 and GATA3 are illustrated in [23] in which TP53, BRCA2 and GATA3 can response to gamma radiation while gene TP53, PIK3CA and GATA3 can regulate the process of neuron apoptotic. Finally, for the Merged-Community, the effect of merge is found not only in the network structure but also in the corresponding known pathway function.

Several methods have been described, but few approaches have considered the overlapping characteristic in modules. We compared our method with a classical clustering method K-means and an advanced method Lemon-tree (Fig. 6). Through GO enrichment analysis, the identified communities perform better than other methods not only the number of GO categories with lower *p*-values but also globally the *p*-values are lower. Two possible reasons are presented to explain why our approach has better performance. The first reason is that our method adds the functional interaction to the relationship between genes which can enhance the interaction between the low-linked and highly functional gene pairs. The second reason is that we construct our framework by considering the overlapping genes among communities. These overlapping genes can play different functions from the combinations of different communities. In addition, the driver genes generated by our approach can accurately distinguish the tumor and normal samples. Furthermore, the prognostic prediction of patients with Kaplan-Meier survival analysis was performed. We found that the significant different survival time is acquired on the identified distinguishing communities.

As a next step, we plan to extend the approach using complementary algorithms developed by other groups, including algorithms that enhance the performance of the module network approach, combining proteomics and metabolomics information as a complement to community construction.

## Conclusion

In summary, it is critical to consider the overlapping characteristic in modules and their communication when analyzing cancer mechanism and process. In order to understand this module features more clearly, we develop a novel coexpressed network analysis framework, which employing the concept of overlapping community to construct and analyze the large biology networks effectively and efficiently. The result shows that our method bridges the gap between communities to find the overlapping genes that provide common functions in different communities. The validity of this method provides more useful information for tumorigenesis mechanism and complex biological network inference, and complements the existing approaches of detecting gene communities.

## Methods

### Datasets and pre-processing

The gene expression and copy number data for six cancer datasets were obtained from The Cancer Genome Atlas (TCGA) [4]. The gene expression values were measured with Illumina HiSeqv2 platform and 2101 tumour vs normal samples were obtained (see Table 1). The copy number data were derived from Affmetrix SNP 6.0 arrays and processed with GISTIC. The clinical data were also obtained from TCGA. Protein-protein interaction (PPI) information from the online database Search Tool for the Retrieval of Interacting Genes (STRING) [44]. Interactions in STRING are uniquely comprehensive coverage and ease of access to both experimental as well as predicted interaction information. From STRING, the known interactions, proved by biological experiments, with a combined *score*>0.4 were retrieved as significant pairs for further analysis. Due to the high heterogeneous data for the six cancers, the Earth mover’s distance [45] was applied to measure the overall diversity between the distributions of a gene’s expression in two classes of samples, normal vs tumor. In our approach, for gene expression and copy number data, the difference between two classes (tumour/normal) in six cancers was analyzed. In gene expression data, we chose the genes with *q*−*values*<0.1 which represents the gene is differentially expressed between class. And for the copy number data, the genes with their *q*−*values*<0.1 were selected. Finally, we combined the filtered gene subsets.

### The initial community clusters

Gibbs sampling can obtain samples from the probability distribution without having to explicitly calculate the value for their narginalizing integeals. It has a good performance to eliminate the non transmission relationship between genes, thus, we applied the Gibbs sampling [46] to infer initial community clusters from a gene expression data matrix. Therefore, due to the approximate distribution of large-scale data sets is multimodal, the mixture of traditional Gibbs sampler may be very poor, the convergence rate will be very slow, and it is difficult to consider the full posterior distribution. the Ganesh can converge the Gibbs sampler on large data sets. It performs non-heuristic reconstruction of gene clusters based on the posterior distribution of the statistical model. we used the “ganesh” software to perform Gibbs sampling [21]. For each cancer, in an expression matrix with *N* genes and *M* samples, iteratively updating the cluster assignment of each gene and samples was performed in 5 times with “ganesh” software (default parameters), which yields 5 partitions. For the *k*_*th*_ Gibbs sampling, we can get a cluster assignment matrix *C*^(*k*)^, i.e. *C*^(*k*)^ is an *N*×*S*_*k*_ matrix where *N* is the number of genes and *S*_*k*_ is the number of clusters from *k*_*th*_ Gibbs sampling. In cluster assignment matrix *C*^(*k*)^, if gene *i* in cluster *S*, $C_{iS}^{k}$=1, otherwise, $C_{iS}^{k}$=0.

### Interaction network construction

#### Edge-weighted bipartite graph network

**Fig. 8:**
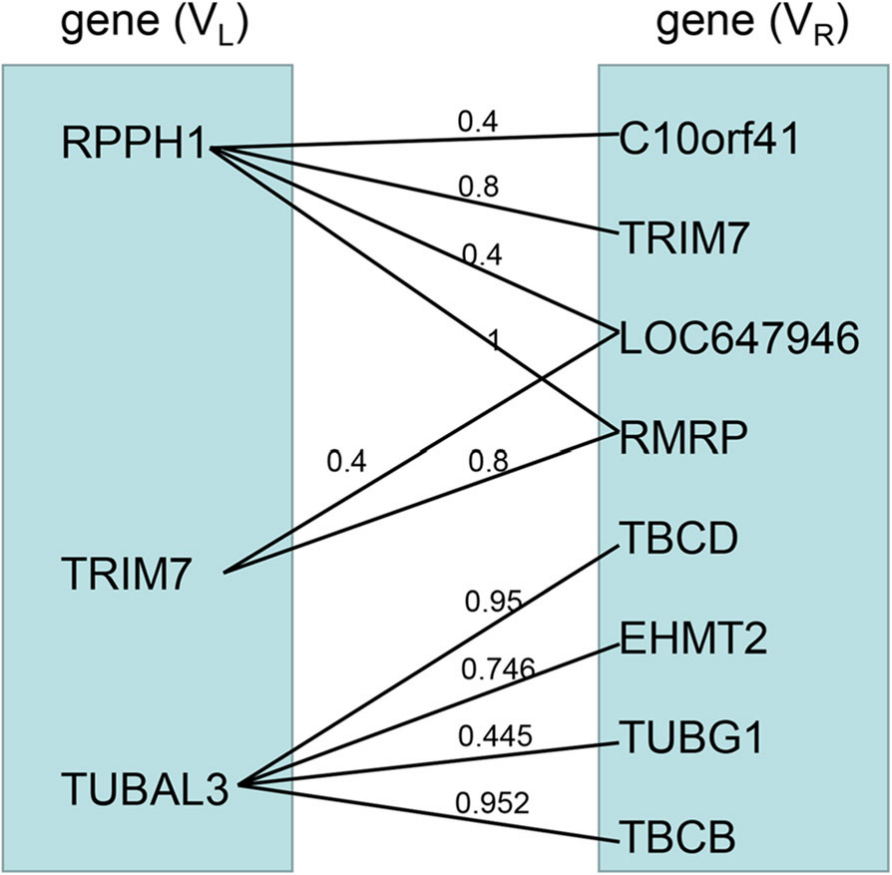
An example of a gene-gene bipartite graph. The gene pairs are represented by an edge-weighted bipartite graph

In this study, the gene-gene interaction network can be regarded as an edge-weighted bipartite graph *G*=(*N*,*E*) for each cluster, where *N* consists of vertices of genes (*V*_*L*_) and genes (*V*_*R*_), *E* denotes the weighted edges between gene. If the constraints between the two genes are met, there is an edge between the two genes. Let *i* be the vertex subscript in *V*_*L*_ and *j* be the vertex subscript in *V*_*R*_, *E*_*ij*_ is an edge connecting between *N*_*i*_ and *N*_*j*_, An example of a gene-gene bipartite graph is shown in Fig. 8.

#### Edge weight calculation

We quantified the connections or weighted edges between gene and gene vertices based on the initial clustering results and STRING database, the algorithm of edge weight calculation is described in Algorithm 1. First, we defined an co-clustering matrix *M*^(*k*)^ for the *k*th Gibbs sampler run. In matrix *M*^(*k*)^, $M_{ij}^{k} = 1$, if gene *i* and gene *j* in a same cluster, and $M_{ij}^{k} = 0$, otherwise. Then, the co-occurrence frequency matrix *M* was obtained from the mean of *M*^(*k*)^ over all *K* runs. In matrix *M*, if *M*_*ij*_>0.4, which means that there is an edge between gene *i* and gene *j*, and the edge weight *E*_*ij*_ was equal to *M*_*ij*_. Finally, the protein-protein interaction information from the STRING database [44] was used to strengthen the correlation between vertices of genes (*V*_*L*_) and genes (*V*_*R*_). Let *P*_*ij*_ be the weight between gene *i* and gene *j* from the STRING database. If the weight between gene *i*(*i*∈*V*_*L*_) and genes *j*(*j*∈*V*_*R*_) was less than *P*_*ij*_, the weight *E*_*ij*_ between gene *i* and gene *j* was updated to *P*_*ij*_.


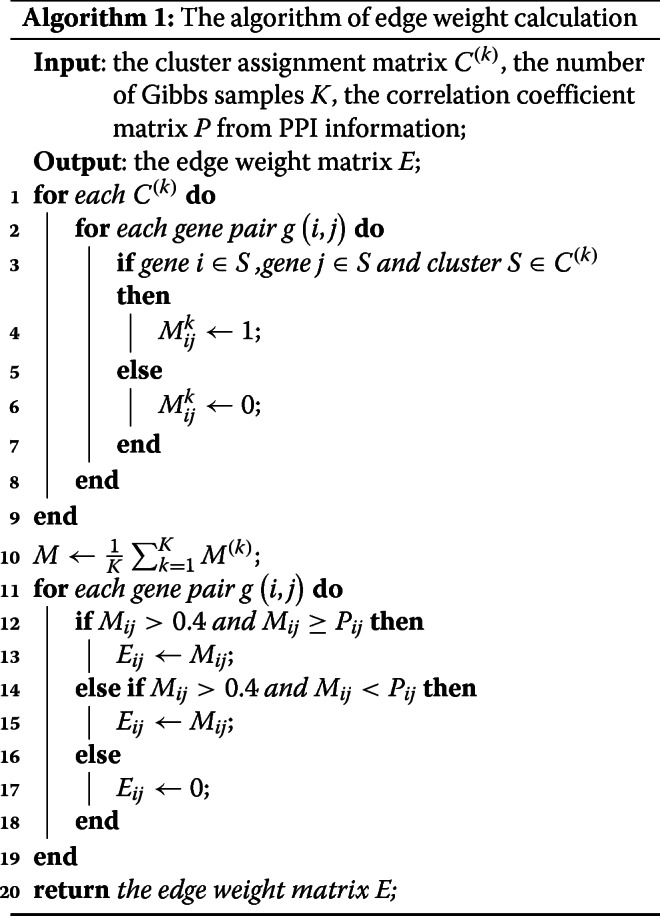


### Identifying high cohesive community

Cohesiveness score (CS) is defined to measure the likelihood of the genes to form a gene community. A greedy growth process were used to construct overlapping gene communities in the gene-gene bipartite graph network G (N, E). The algorithm consists of five steps. First of all, the gene with the maximal degree (largest amount of connections) was chosen as the initial node, and a cohesive community was constructed by the greedy procedure. At the end of each growth process, the algorithm considered all genes that have not been included in any gene community to date, and select the genes with the largest number of connections as the next seed again. When there are no genes remaining to be considered, the whole process ends. The step-by-step description of the greedy growth procedure starting from the seed node *s*_0_ is as follows: Step 1: let *S*_0_={*s*_0_} and set the greedy step *t*=0. Step 2: calculate the cohesiveness value *CS*(*S*_*t*_) of *S*_*t*_ and let *S*_*t*+1_=*S*_*t*_. Step 3: for every external gene *s* incident on at least one boundary edge with *S*_*t*_, calculate the cohesiveness value $CS (S_{t} \bigcup \{s\})$. If $CS (S_{t}\bigcup \{s\}) > CS (S_{t+1})$, let $S_{t+1} = S_{t}\bigcup \{s\}$. Step 4: for every internal gene s incident on at least one boundary edge, calculate the cohesiveness value *CS*(*S*_*t*_∖{*s*}). If *CS*(*S*_*t*_∖{*s*})>*CS*(*S*_*t*+1_), let *S*_*t*+1_=*S*_*t*_∖{*s*}. Step 5: if *S*_*t*_≠*S*_*t*+1_, increase *t* and return to step2. Otherwise, *S*_*t*_ is regarded as a locally optimal cohesive community. The algorithm for identifying high cohesive community is described in Additional file 1: Algorithm S1. The cohesiveness score is defined as follows,

1 $$ {}CS (S) = \sum_{i\in S}\frac{\sum_{i\in S} \sum_{j\in S}^{i \neq j} E_{ij}}{\sum_{i\in S} \sum_{j\in S}^{i \neq j} E_{ij}+\sum_{i\in S} \sum_{j\notin S} E_{ij}+p\left | S \right |}  $$

Where *i* is the gene in cluster *S*, *E*_*ij*_ represents the weight between the gene *i* and gene *j*, *p*|*S*| is a penalty term to take into account undetected interactions in genetic networks. The default setting of *p*|*S*| is 0.2.

### Identification of community inference models

**Fig. 9:**
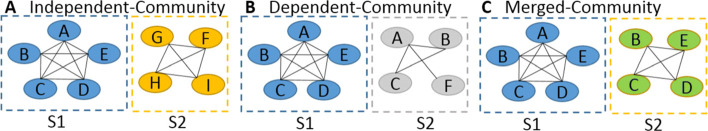
Overview of three overlap situations for which our method was processed. In this network, the same letter represents the same node, and different letters represent different nodes. **a** Independent-Community model: the overlap score of two gene communities S1 and S2, *ω*(*S*1,*S*2)=0. **b** Dependent-Community model: *ω*(*S*1,*S*2)<*ω* and *ω*(*S*1,*S*2)>0. *ω* is a positive thereshold. **c** Merged-Community model: *ω*(*S*1,*S*2)≥*ω*. *ω* is a positive thereshold

As we consider the value of overlapping score among the gene communities, we propose three models in our framework. Independent-Community (IC) model detects communities that act independently on cancer. Dependent community (DC) model can communicate with other communities by searching for such gene community possessing certain gene with multiple biological functions. Merged-Community (MC) model contains most common genes in two modules to play co-function in one module, which is necessary to community detection for community structure stabilization and community diversity elimination. The overview of three models from our approach is shown in Fig. 9.

The overlap score is defined to measure the proportion of common genes in two communities (*S*1 and *S*2). The overlap score *ω* between two communities is calculated as follows,

2 $$ \omega (S1,S2)=\frac{\left | S1\cap S2 \right |^{2}}{\left | S1 \right |\left | S2 \right |}   $$

Given a threshold *ω*, community *S*1 and community *S*2 with high cohesiveness score (CS). If the overlap score is 0, we call it an independent community model. The community is called a dependent community when *ω*(*S*1,*S*2)<*ω*, and community *S*1 and community *S*2 are reserved as two dependent communities. Otherwise, it is called a Merged-Community module, and community *S*1 and community *S*2 are merged into one community (*S*). This threshold *ω* can be determined based on the results of Gene Ontology (GO) enrichment. In this study, we set *ω* = 0.8.

### Survival analysis

The association of gene expression level with the patient survival in each community was analyzed by Cox proportional hazard model [47], and it was performed using the function *coxph* (R package survival). Only patients with fully characterized tumors and with at least 30 days of overall survival (OS) were included in this study. Cancer samples were divided into high-expression group and low-expression group based on the average expression value of each community. The difference between these two classes of patients was acquired by using the Kaplan-Meier estimator and log-rank method.

### Comparison benchmark for Lemon-Tree, K-Means and our method

Gene Ontology (GO) enrichment was performed using the BiNGO Java library [48]. The comparison of the K-Means [49], Lemon-Tree [21] and our approach was conducted by computing the *p*-value and corrected *p*-value. Enriched ontological terms with the corrected *p*-value <0.05 were selected. For these three methods, the K-Means method ignores that there is often a lot of noise in biological data and cannot automatically determine clustering parameters. Lemon-Tree combines multiple omics data to use the idea of gene network reconstruction to infer the initial modules, but only considers independent modules. However, in real biological networks, gene modules may have overlapping characteristics. Our method takes this characteristics into account.

### In silico validation

For each cancer, we selected the regulating genes. Classification of normal tumor samples using the SVM model (https://cran.r-project.org/web/packages/kernlab/index.html) [50–52]. The area under curve (AUC) and Receiver Operating Characteristic (ROC) curves were employed to measure the performance by a cross-validation method (k-fold cross-validation, k = 10). We adopted the following parameters: type="C-bsvc", C (cost of constraints violation)=10.

## Supplementary Information

**Additional file 1** **Figure S1**. The rest of Independent-Communities networks in community network, enriched with the known Gene Ontology (GO) terms. The node and edge represent genes and interaction in community.
**Figure S2**. Functional pathway in ClueGO. It presents the genes and information related to their associated genes. The bars represent the number of the genes from Community NO.121, 126, 172 found associated with the pathway. The different color of the bars denotes diverse pathway types.
**Table S1**. Number of mutated samples for each subtype for each community. This table supplements the discussion of community identified in Independent-Community model. In Table 1 in S1 Text we show the number of mutated samples for each community for each subtype. Figure 3C in S1 Text shows the distribution for each gene. The total number of mutated samples for 4 subtypes are shown in the first row. For each community, we computed the number of samples with mutations in at least one gene in the community (followed by the number of mutated samples for each gene in the community). * indicates the significance of subtype enrichment relative to the overall mutations of a given community (or a gene) across all the subtypes. (*** for p < 0.01, ** for p < 0.05, and * for p < 0.1).
**Table S2**. The list of AUC, regulation score and module about top-10 driver genes in each cancer.

**Additional file 2** **Table S3**. The model types of identified communities in our method.

**Additional file 3** **Table S4**. Detailed information about the enriched pathways and their associated genes.

**Additional file 4** **Table S5**. The detailed description for the communities merging.

## Data Availability

All data used in this manuscript were downloaded from public repositories. The clinical data, gene expression data and copy number data for six cancer datasets were available in The Cancer Genome Atlas (https://portal.gdc.cancer.gov/). The protein-protein interaction information can be accessed with the https://string-db.org/.

## Abbreviations

NGS: next generation sequencing
TCGA: The Cancer Genome Atlas
COSMIC: Catalogue Of Somatic Mutations In Cancer
CNV: copy number data
PPI: protein-protein interaction
IC: Independent-Community
DC: Dependent-Community
MC: Merged-Community
CS: Cohesiveness score
GO: Gene Ontology
OS: overall survival
AUC: area under curve
ROC: Receiver Operating Characteristic
BP: biology process
CC: cellular component
MF: molecular functions

## References

[1] Zhang W, Wang SL. An integrated framework for identifying mutated driver pathway and cancer progression. IEEE/ACM Trans Comput Biol Bioinforma. 2019. 10.1109/TCBB.2017.2788016. 10.1109/TCBB.2017.2788016 29990286

[2] Huang D-S, Zhang L, Han K, Deng S, Yang K, Zhang H. Prediction of Protein-Protein Interactions Based on Protein-Protein Correlation Using Least Squares Regression. Curr Protein Pept Sci. 2014. 10.2174/1389203715666140724084019. 10.2174/1389203715666140724084019 25059329

[3] Shendure J, Ji H. Next-generation DNA sequencing. 2008. 10.1038/nbt1486. 10.1038/nbt1486 18846087

[4] Tomczak K, Czerwińska P, Wiznerowicz M. The Cancer Genome Atlas (TCGA): An immeasurable source of knowledge. 2015. 10.5114/wo.2014.47136. 10.5114/wo.2014.47136 PMC4322527 25691825

[5] Forbes S, Clements J, Dawson E, Bamford S, Webb T, Dogan A, Flanagan A, Teague J, Wooster R, Futreal PA (2006). Cosmic 2005. Br J Cancer.

[6] You ZH, Lei YK, Gui J, Huang DS, Zhou X. Using manifold embedding for assessing and predicting protein interactions from high-throughput experimental data. Bioinformatics. 2010. 10.1093/bioinformatics/btq510. 10.1093/bioinformatics/btq510 PMC3025743 20817744

[7] Xia JF, Zhao XM, Song J, Huang DS. APIS: Accurate prediction of hot spots in protein interfaces by combining protrusion index with solvent accessibility. BMC Bioinformatics. 2010. 10.1186/1471-2105-11-174. 10.1186/1471-2105-11-174 PMC2874803 20377884

[8] Tao H, Min J, Kong X, Cai YD (2012). Dysfunctions associated with methylation, microrna expression and gene expression in lung cancer. PLoS ONE.

[9] Lu X, Wang X, Ding L, Gao Y, He K. frdriver: A functional region driver identification for protein sequence. IEEE/ACM Transactions on Computational Biology and Bioinformatics. 2020. 10.1109/TCBB.2020.3020096. 10.1109/TCBB.2020.3020096 32870797

[10] Nepusz T, Petróczi A, Négyessy L, Bazsó F. Fuzzy communities and the concept of bridgeness in complex networks. Phys Rev E Stat Nonlinear Soft Matter Phys. 2008. 10.1103/PhysRevE.77.016107. 10.1103/PhysRevE.77.016107 18351915

[11] Althammer S, Pagès A, Eyras E. Predictive models of gene regulation from high-throughput epigenomics data. Comp Funct Genomics. 2012. 10.1155/2012/284786. 10.1155/2012/284786 PMC3424690 22924024

[12] Lu X, Qian X, Li X, Miao Q, Peng S (2019). Dmcm: a data-adaptive mutation clustering method to identify cancer-related mutation clusters. Bioinformatics.

[13] Lu X, Li X, Liu P, Qian X, Miao Q, Peng S (2018). The integrative method based on the module-network for identifying driver genes in cancer subtypes. Molecules.

[14] Friedman N (2004). Inferring cellular networks using probabilistic graphical models. Science.

[15] Lu X, Lu J, Liao B, Li X, Qian X, Li K. Driver pattern identification over the gene co-expression of drug response in ovarian cancer by integrating high throughput genomics data. Sci Rep. 2017. 10.1038/s41598-017-16286-5. 10.1038/s41598-017-16286-5 PMC5700962 29170526

[16] Eisen MB, Spellman PT, Brown PO, Botstein D (1998). Cluster analysis and display of genome-wide expression patterns. Proc Natl Acad Sci.

[17] Joshi A, Van de Peer Y, Michoel T (2008). Analysis of a gibbs sampler method for model-based clustering of gene expression data. Bioinformatics.

[18] Dahl DB (2006). Model-based clustering for expression data via a dirichlet process mixture model. Bayesian inference for gene expression and proteomics.

[19] Michoel T, De Smet R, Joshi A, Van de Peer Y, Marchal K (2009). Comparative analysis of module-based versus direct methods for reverse-engineering transcriptional regulatory networks. BMC Syst Biol.

[20] Roy S, Lagree S, Hou Z, Thomson JA, Stewart R, Gasch AP (2013). Integrated module and gene-specific regulatory inference implicates upstream signaling networks. PLoS Comput Biol.

[21] Bonnet E, Calzone L, Michoel T (2015). Integrative multi-omics module network inference with lemon-tree. Plos Comput Biol.

[22] Wang Z, Zhang D, Zhou X, Yang D, Yu Z, Yu Z. Discovering and profiling overlapping communities in location-based social networks. IEEE Trans Syst Man Cybern Syst. 2014. 10.1109/TSMC.2013.2256890.

[23] Hou JP, Ma J. DawnRank: Discovering personalized driver genes in cancer. Genome Med. 2014. 10.1186/s13073-014-0056-8. 10.1186/s13073-014-0056-8 PMC4148527 25177370

[24] Nolan D, Ginsberg M, Israely E, Palikuqi B, Poulos MG, James D, Ding BS, Schachterle W, Liu Y, Rosenwaks Z (2013). Molecular signatures of tissue-specific microvascular endothelial cell heterogeneity in organ maintenance and regeneration. Dev Cell.

[25] Shannon P, Markiel A, Ozier O, Baliga NS, Wang JT, Ramage D, Amin N, Schwikowski B, Ideker T. Cytoscape: A software Environment for integrated models of biomolecular interaction networks. Genome Res. 2003. 10.1101/gr.1239303. 10.1101/gr.1239303 PMC403769 14597658

[26] Rives AW, Galitski T. Proc Natl Acad Sci U S A. 2003; 100(3):1128–33. 10.1073/pnas.0237338100. 10.1073/pnas.0237338100 PMC298738 12538875

[27] D’Haeseleer P (2005). How does gene expression clustering work?. Nat Biotechnol.

[28] Spirin V, Mirny LA. Proc Natl Acad Sci U S A. 2003; 100(21):12123–8. 10.1073/pnas.2032324100. 10.1073/pnas.2032324100 PMC218723 14517352

[29] Heagerty PJ, Lumley T, Pepe MS. Time-dependent ROC curves for censored survival data and a diagnostic marker. Biometrics. 2000. 10.1111/j.0006-341X.2000.00337.x. 10.1111/j.0006-341x.2000.00337.x 10877287

[30] Hofland J, Delhanty PJ, Steenbergen J, Hofland LJ, van Koetsveld PM, van Nederveen FH, de Herder WW, Feelders RA, de Jong FH (2012). Melanocortin 2 receptor-associated protein (mrap) and mrap2 in human adrenocortical tissues: regulation of expression and association with acth responsiveness. J Clin Endocrinol.

[31] Daves MH, Hilsenbeck SG, Lau CC, Man TK. Meta-analysis of multiple microarray datasets reveals a common gene signature of metastasis in solid tumors. BMC Med Genomics. 2011. 10.1186/1755-8794-4-56. 10.1186/1755-8794-4-56 PMC3212952 21736749

[32] Zhang X, Sheng J, Zhang Y, Tian Y, Zhu J, Luo N, Xiao C, Li R. Overexpression of SCAMP3 is an indicator of poor prognosis in hepatocellular carcinoma. Oncotarget. 2017. 10.18632/oncotarget.22665. 10.18632/oncotarget.22665 PMC5752518 29312605

[33] Aoh QL, Castle AM, Hubbard CH, Katsumata O, Castle JD. SCAMP3 Negatively Regulates Epidermal Growth Factor Receptor Degradation and Promotes Receptor Recycling. Mol Biol Cell. 2009. 10.1091/mbc.e08-09-0894. 10.1091/mbc.E08-09-0894 PMC2655259 19158374

[34] Balasubramani A, Larjo A, Bassein JA, Chang X, Hastie RB, Togher SM, Lähdesmäki H, Rao A (2015). Cancer-associated asxl1 mutations may act as gain-of-function mutations of the asxl1–bap1 complex. Nat Commun.

[35] Nii K, Tokunaga Y, Liu D, Zhang X, Nakano J, Ishikawa S, Kakehi Y, Haba R, Yokomise H (2014). Overexpression of g protein-coupled receptor 87 correlates with poorer tumor differentiation and higher tumor proliferation in non-small-cell lung cancer. Mol Clin Oncol.

[36] Zhang ZF, Zhang HR, Zhang QY, Lai SY, Feng YZ, Zhou Y, Zheng SR, Shi R, Zhou JY. High expression of TMEM40 is associated with the malignant behavior and tumorigenesis in bladder cancer. J Transl Med. 2018. 10.1186/s12967-017-1377-3. 10.1186/s12967-017-1377-3 PMC5775579 29351801

[37] Ciró M, Prosperini E, Quarto M, Grazini U, Walfridsson J, McBlane F, Nucifero P, Pacchiana G, Capra M, Christensen J, Helin K. ATAD2 is a novel cofactor for MYC, overexpressed and amplified in aggressive tumors. Cancer Res. 2009. 10.1158/0008-5472.CAN-09-2131. 10.1158/0008-5472.CAN-09-2131 19843847

[38] Wang L, Jia YP, Jiang ZY, Gao W, Wang BQ. FSCN1 is upregulated by SNAI2 and promotes epithelial to mesenchymal transition in head and neck squamous cell carcinoma. Cell Biol Int. 2017. 10.1002/cbin.10786. 10.1002/cbin.10786 28488774

[39] Patel V, Adhil M, Bhardwaj T, Talukder AK (2015). Big data analytics of genomic and clinical data for Diagnosis and Prognosis of Cancer. 2015 2nd International Conference on Computing for Sustainable Global Development (INDIACom).

[40] Hou Y, Gao B, Li G, Su Z. MaxMIF: A New Method for Identifying Cancer Driver Genes through Effective Data Integration. Adv Sci. 2018. 10.1002/advs.201800640. 10.1002/advs.201800640 PMC6145398 30250803

[41] Wu K, Li Z, Cai S, Tian L, Chen K, Wang J, Hu J, Sun Y, Li X, Ertel A (2013). Eya1 phosphatase function is essential to drive breast cancer cell proliferation through cyclin d1. Cancer Res.

[42] Blevins MA, Towers CG, Patrick AN, Zhao R, Ford HL (2015). The six1-eya transcriptional complex as a therapeutic target in cancer. Expert Opin Ther Targets.

[43] Rives AW, Galitski T. Modular organization of cellular networks. Proc Natl Acad Sci. 2003. 10.1073/pnas.0237338100. 10.1073/pnas.0237338100 PMC298738 12538875

[44] Szklarczyk D, Franceschini A, Wyder S, Forslund K, Heller D, Huerta-Cepas J, Simonovic M, Roth A, Santos A, Tsafou KP (2014). String v10: protein–protein interaction networks, integrated over the tree of life. Nucleic Acids Res.

[45] Nabavi S, Schmolze D, Maitituoheti M, Malladi S, Beck AH. EMDomics: A robust and powerful method for the identification of genes differentially expressed between heterogeneous classes. Bioinformatics. 2016. 10.1093/bioinformatics/btv634. 10.1093/bioinformatics/btv634 PMC4743632 26515818

[46] Joshi A, Van de peer Y, Michoel T. Analysis of a Gibbs sampler method for model-based clustering of gene expression data. Bioinformatics. 2008. 10.1093/bioinformatics/btm562. 10.1093/bioinformatics/btm562 18033794

[47] Therneau TM. A Package for Survival Analysis in S. Version 2.38. 2015. CRAN website - http://cran.r-project.org/package=survival. Accessed 12 June 2015.

[48] Maere S, Heymans K, Kuiper M. BiNGO: a Cytoscape Plugin to Assess Overrepresentation of Gene Ontology Categories in Biological Networks: Oxford University Press; 2005, pp. 3448–9. 10.1093/bioinformatics/bti551. 10.1093/bioinformatics/bti551 15972284

[49] Hartigan JA, Wong MA. Algorithm AS 136: A K-Means Clustering Algorithm. Appl Stat. 2006. 10.2307/2346830.

[50] Karatzoglou A, Smola A, Hornik K, Zeileis A. kernlab - An S4 Package for Kernel Methods in R. J Stat Softw. 2015. 10.18637/jss.v011.i09.

[51] Clausel M, Grégoire G (2014). Practical Session: Introduction to R. EAS Publ Ser.

[52] Karatzoglou A, Smola A, Hornik K, et al.Kernlab: Kernel-based machine learning lab. Version 0.9. 2016. CRAN website - https://cran.r-project.org/web/packages/kernlab.

