# Supplementary material for: A novel method to identify gene interaction patterns
Source: BMC Genomics. 2021 Jun 10;22(Suppl 1):436. doi: 10.1186/s12864-021-07628-9 (PMC8194229; doi:10.1186/s12864-021-07628-9)

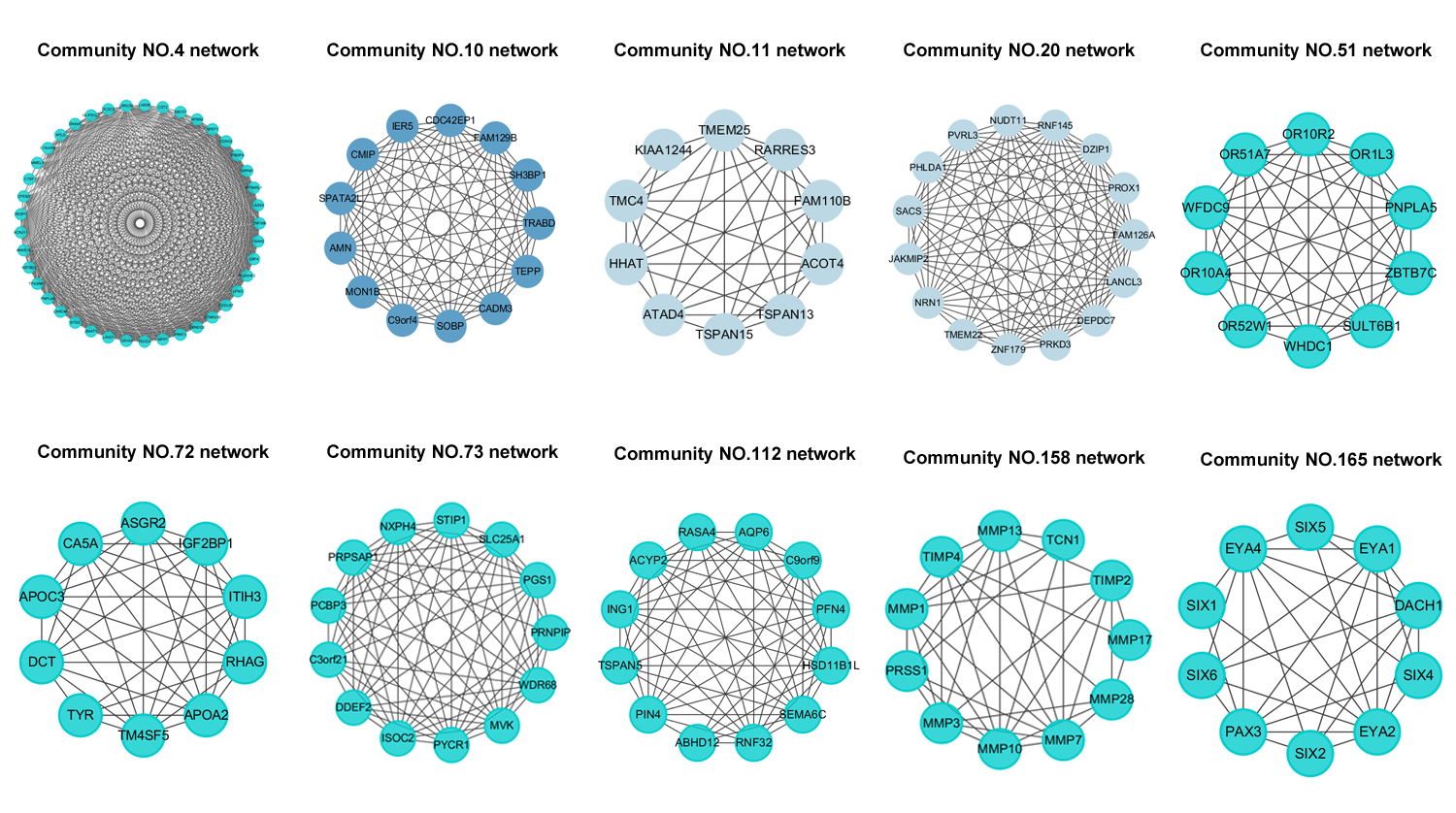


**Fig. S1**. The rest of Independent-Communities networks in community network, enriched with the known Gene Ontology(GO) terms. The node and edge represent genes and interaction in community.


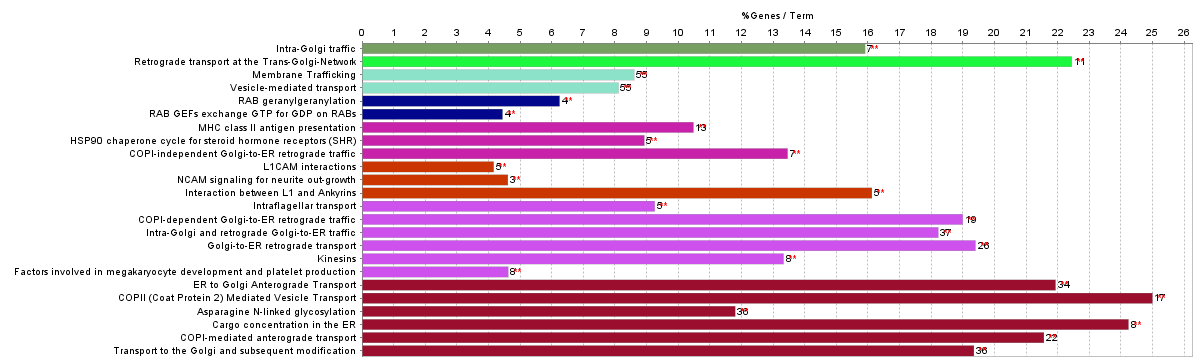


**Fig. S2**. Functional pathway in ClueGO. It presents the genes and information related to their associated genes. The bars represent the number of the genes from Community NO.121, 126, 172 found associated with the pathway. The different color of the bars denotes diverse pathway types.

**Table S1. Number of mutated samples for each subtype for each community.**

|  | **All** | **lumA(215)** | **lumB(122)** | **Basal(92)** | **Her2(55)** |
| --- | --- | --- | --- | --- | --- |
| Community NO.4(LRIG1,PDCD4) | 330 | 120 | 97(***) | 68 | 45(***) |
| Community NO.10  (FAM129B,MON1B) | 340 | 166(***) | 87 | 55 | 32 |
| Community NO.20  (PROX1,PRKD3,PHLDA1) | 376 | 152 | 106(***) | 76 | 42 |
| Community NO.112  (ING1,HSD11B1) | 388 | 165 | 107(***) | 72 | 44 |
| Community NO.141  (PKP2,KRT1) | 158 | 42 | 40 | 55(***) | 21 |
| Community NO.165  (EYA1,EYA2,EYA4) | 364 | 136 | 104(***) | 73 | 47(***) |

*This table supplements the discussion of community identified in Independent-Community model. In Table 1 in S1 Text we show the number of mutated samples for each community for each subtype. Figure 3C in S1 Text shows the distribution for each gene. The total number of mutated samples for 4 subtypes are shown in the first row. For each community, we computed the number of samples with mutations in at least one gene in the community (followed by the number of mutated samples for each gene in the community). * indicates the significance of subtype enrichment relative to the overall mutations of a given community (or a gene) across all the subtypes. (*** for p < 0.01, ** for p < 0.05, and * for p < 0.1).*

**Table S2. The list of AUC, regulation score and module about top-10 driver genes in each cancer**

| **Cancer Type** | **TCGA ID Data** | **Driver Genes** | **Module Number** | **Regulation Score** | **AUC** |
| --- | --- | --- | --- | --- | --- |
| Kidney Chromophobe | KICH | MRAP2 | 145 | 18.209 | 0.985 |
| Kidney Chromophobe | KICH | EDARADD | 275 | 24.692 | 0.954 |
| Kidney Chromophobe | KICH | OXGR1 | 136 | 24.759 | 0.954 |
| Kidney Chromophobe | KICH | USP51 | 23 | 67.044 | 0.908 |
| Kidney Chromophobe | KICH | ZNF41 | 20 | 12.592 | 0.908 |
| Kidney Chromophobe | KICH | LANCL3 | 237 | 22.200 | 0.877 |
| Kidney Chromophobe | KICH | SLC25A19 | 16 | 26.795 | 0.815 |
| Kidney Chromophobe | KICH | CYSLTR2 | 114 | 14.171 | 0.769 |
| Kidney Chromophobe | KICH | IGSF1 | 104 | 38.579 | 0.754 |
| Kidney Chromophobe | KICH | MTHFD1L | 277 | 12.817 | 0.754 |
| Bladder Urothelial Carcinoma | BLCA | FGFR1 | 6 | 84.373 | 1 |
| Bladder Urothelial Carcinoma | BLCA | ADCK5 | 32 | 67.474 | 0.975 |
| Bladder Urothelial Carcinoma | BLCA | ATAD2 | 133 | 41.459 | 0.969 |
| Bladder Urothelial Carcinoma | BLCA | RBPMS | 81 | 34.433 | 0.963 |
| Bladder Urothelial Carcinoma | BLCA | RHPN1 | 28 | 67.836 | 0.941 |
| Bladder Urothelial Carcinoma | BLCA | HIC1 | 174 | 37.393 | 0.923 |
| Bladder Urothelial Carcinoma | BLCA | DPYSL2 | 6 | 33.827 | 0.920 |
| Bladder Urothelial Carcinoma | BLCA | GLIPR2 | 231 | 51.288 | 0.892 |
| Bladder Urothelial Carcinoma | BLCA | KCNK9 | 37 | 49.740 | 0.880 |
| Bladder Urothelial Carcinoma | BLCA | LY6E | 90 | 32.282 | 0.855 |
| Breast invasive carcinoma cancer | BRCA | PEX19 | 40 | 23.348 | 0.969 |
| Breast invasive carcinoma cancer | BRCA | LCE2D | 15 | 113.855 | 0.959 |
| Breast invasive carcinoma cancer | BRCA | PSMD4 | 6 | 26.168 | 0.943 |
| Breast invasive carcinoma cancer | BRCA | TNNI1 | 133 | 27.894 | 0.892 |
| Breast invasive carcinoma cancer | BRCA | SCAMP3 | 12 | 23.216 | 0.866 |
| Breast invasive carcinoma cancer | BRCA | CNIH3 | 23 | 41.737 | 0.851 |
| Breast invasive carcinoma cancer | BRCA | MUC1 | 195 | 30.408 | 0.835 |
| Breast invasive carcinoma cancer | BRCA | CD48 | 70 | 62.065 | 0.789 |
| Breast invasive carcinoma cancer | BRCA | LAD1 | 176 | 32.105 | 0.773 |
| Breast invasive carcinoma cancer | BRCA | CDC73 | 131 | 59.787 | 0.768 |
| Colon adenocarcinoma | COAD | ASXL1 | 396 | 48.993 | 0.929 |
| Colon adenocarcinoma | COAD | PLCG1 | 275 | 22.100 | 0.903 |
| Colon adenocarcinoma | COAD | DDX27 | 158 | 63.483 | 0.901 |
| Colon adenocarcinoma | COAD | SNHG11 | 129 | 27.688 | 0.879 |
| Colon adenocarcinoma | COAD | HCK | 228 | 160.939 | 0.877 |
| Colon adenocarcinoma | COAD | PRPF6 | 129 | 30.836 | 0.875 |
| Colon adenocarcinoma | COAD | STX16 | 159 | 21.182 | 0.862 |
| Colon adenocarcinoma | COAD | SPG20 | 305 | 29.430 | 0.841 |
| Colon adenocarcinoma | COAD | KIFC2 | 22 | 94.267 | 0.821 |
| Colon adenocarcinoma | COAD | CDCA2 | 328 | 65.171 | 0.817 |
| Esophageal carcinoma | ESCA | SLC4A4 | 23 | 52.020 | 0.932 |
| Esophageal carcinoma | ESCA | GPR87 | 61 | 23.977 | 0.919 |
| Esophageal carcinoma | ESCA | EREG | 124 | 55.667 | 0.919 |
| Esophageal carcinoma | ESCA | DSC3 | 39 | 44.682 | 0.905 |
| Esophageal carcinoma | ESCA | DSG3 | 95 | 84.003 | 0.878 |
| Esophageal carcinoma | ESCA | CNTN4 | 7 | 20.290 | 0.865 |
| Esophageal carcinoma | ESCA | TWIST1 | 123 | 35.348 | 0.837 |
| Esophageal carcinoma | ESCA | MYL9 | 50 | 35.502 | 0.757 |
| Esophageal carcinoma | ESCA | ADH1C | 56 | 18.956 | 0.730 |
| Esophageal carcinoma | ESCA | RNF150 | 53 | 88.520 | 0.689 |
| Head and Neck squamous cell carcinoma | HNSC | ATP6V0D2 | 141 | 38.139 | 0.958 |
| Head and Neck squamous cell carcinoma | HNSC | MED30 | 69 | 24.713 | 0.957 |
| Head and Neck squamous cell carcinoma | HNSC | TMEM40 | 37 | 136.603 | 0.955 |
| Head and Neck squamous cell carcinoma | HNSC | SNAI2 | 94 | 80.236 | 0.935 |
| Head and Neck squamous cell carcinoma | HNSC | ACTL6A | 91 | 30.896 | 0.933 |
| Head and Neck squamous cell carcinoma | HNSC | SLURP1 | 39 | 153.772 | 0.904 |
| Head and Neck squamous cell carcinoma | HNSC | TM4SF19 | 207 | 24.920 | 0.900 |
| Head and Neck squamous cell carcinoma | HNSC | PRKAR2A | 95 | 84.628 | 0.893 |
| Head and Neck squamous cell carcinoma | HNSC | SETMAR | 173 | 19.802 | 0.893 |
| Head and Neck squamous cell carcinoma | HNSC | SATB1 | 229 | 31.989 | 0.872 |

**Algorithm S1. The algorithm of overlapping community identification**


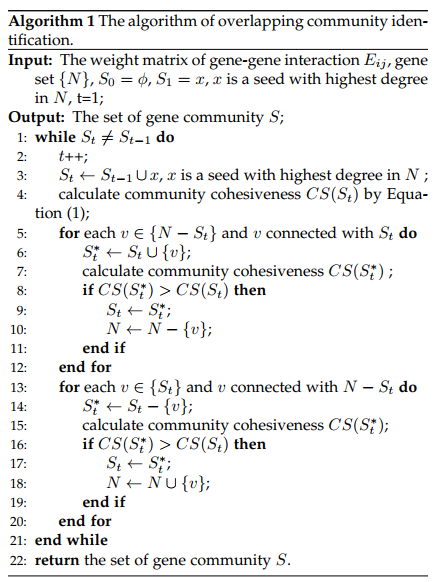

Supplement: Supplementary file 1 — Additional file 1 Figure S1. The rest of Independent-Communities networks in community network, enriched with the known Gene Ontology (GO) terms. The node and edge represent genes and interaction in community. Figure S2. Functional pathway in ClueGO. It presents the genes and information related to their associated genes. The bars represent the number of the genes from Community NO.121, 126, 172 found associated with the pathway. The different color of the bars denotes diverse pathway types. Table S1. Number of mutated samples for each subtype for each community. This table supplements the discussion of community identified in Independent-Community model. In Table 1 in S1 Text we show the number of mutated samples for each community for each subtype. Figure 3C in S1 Text shows the distribution for each gene. The total number of mutated samples for 4 subtypes are shown in the first row. For each community, we computed the number of samples with mutations in at least one gene in the community (followed by the number of mutated samples for each gene in the community). * indicates the significance of subtype enrichment relative to the overall mutations of a given community (or a gene) across all the subtypes. (*** for p < 0.01, ** for p < 0.05, and * for p < 0.1). Table S2. The list of AUC, regulation score and module about top-10 driver genes in each cancer. [file 12864_2021_7628_MOESM1_ESM.docx]
